# Expression and Promoter Methylation of the Genes Encoding the Mitochondrial and Cytosolic Forms of Fumarase in Sunflower (*Helianthus annuus* L.) Leaves Depending on Light Regime and Salinity

**DOI:** 10.3390/cimb48050513

**Published:** 2026-05-15

**Authors:** Oksana V. Sazonova, Dmitry N. Fedorin, Alexander T. Eprintsev, Abir U. Igamberdiev

**Affiliations:** 1 Department of Biochemistry and Cell Physiology, Voronezh State University, 394018 Voronezh, Russia; oksana_ragik@mail.ru (O.V.S.); rybolov@mail.ru (D.N.F.); bc366@bio.vsu.ru (A.T.E.); 2 Laboratory for Biotechnological and Molecular Genetic Research of Plants, Voronezh State Agrarian University, 394087 Voronezh, Russia; 3 Department of Biology, Memorial University of Newfoundland, 45 Arctic Avenue, St. John’s, NL A1C 5S7, Canada

**Keywords:** sunflower (*Helianthus annuus* L.), DNA methylation, fumarase, phytochrome, salt stress, tricarboxylic acid cycle

## Abstract

The expression of two genes, *Fum1* and *Fum2*, encoding the mitochondrial and cytosolic forms of fumarase (EC 4.2.1.2); the methylation of individual CpGs of their promoters; and fumarase activity were studied in sunflower (*Helianthus annuus* L.) leaves depending on irradiation and salinity. Fumarase activity was twice as high in darkness compared to irradiation by white light and red light, while far-red light applied after darkness or after red light reverted the activity to the values in darkness, which indicates the involvement of phytochrome. Using qRT-PCR, it was demonstrated that this corresponded to the pattern of expression of the *Fum1* gene, while the expression of the *Fum2* gene was higher upon irradiation by white and red light, and lower in darkness and under far-red light. Under the application of 150 mM NaCl for 1, 3, 6, 12, and 24 h, fumarase activity increased fivefold from the start of incubation to 6 h, and then decreased after 12 h. These changes were associated with the transcriptional regulation of the *Fum1* and *Fum2* genes. Changes in the methylation status of the analyzed CpGs in their gene promoters, detected via semi-quantitative methylation-specific PCR, were associated with differences in their expression. The higher methylation levels of the analyzed CpGs in the *Fum1* gene promoter under different light conditions and in the *Fum2* gene promoter under salinity corresponded to low levels of their transcripts in sunflower leaves. It is suggested that the mitochondrial and cytosolic forms of fumarase are regulated by light and salinity at the gene expression level, presumably through changes in the methylation status of individual CpGs in their promoters.

## 1. Introduction

Fumarase (fumarate hydratase; EC 4.2.1.2) catalyzes the reaction of the interconversion of malate and fumarate. It is one of the enzymes of the tricarboxylic acid (TCA) cycle. It has a dual localization in mitochondria and the cytosol. The cytosolic isoform has been characterized in Arabidopsis [1] and in maize [2]. In some plants, separate genes encode the mitochondrial and cytosolic forms of fumarase, while in poplar [3] and tomato [4], only one fumarase gene is involved, which can undergo alternative splicing, as in the case of the chloroplast and cytosolic forms of glycerate kinase [5]. Different functions have been attributed to the cytosolic form of fumarase, including its participation in nitrogen accumulation and cold acclimation [6], temperature acclimation [7], the regulation of Fe and Cu incorporation into metalloproteins [8], and the utilization of succinate formed in the glyoxylate cycle [2]. It has been demonstrated that cytosolic fumarase, by moving its protein to the nucleus, can participate in the cellular response to DNA double-strand breaks [9].

Sunflower (*Helianthus annuus* L.) accumulates fumaric acid in both shoots and roots, where its concentration exceeds the amounts of malate and citrate [10]. Fumarate can play multiple roles in plants, including carbon storage [11,12,13], the regulation of malate conversion during light–dark transitions [14,15], and as an intermediate of gluconeogenesis [16,17].

Epigenetic mechanisms—particularly DNA methylation, which represents changes in DNA that do not affect its primary structure—can play an important role in the control of gene expression [18], thereby providing rapid adaptive responses to stress [19]. Under high salinity conditions, DNA methylation is particularly common and is accompanied by changes in gene expression that contribute to increased salt tolerance in plants [20]. The addition of a methyl group to a nucleotide sequence is critical for the regulation of gene function and the response to abiotic stress, especially in plants with complex genomes [21]. In addition to DNA methylation, well-known epigenetic mechanisms that ensure plant adaptation to stress include histone modifications and non-coding RNAs.

In previous studies conducted by our group, we established the participation of cytosolic fumarase in gluconeogenesis in maize scutellum [2] and sunflower cotyledons [22]. We revealed the differential regulation of the two isoforms under hypoxic conditions in maize leaves [23]. We demonstrated that phytochrome mediated the light regulation of fumarase in Arabidopsis [24] and that cryptochrome is involved in the regulation of mitochondrial fumarase in maize [25]. We also established that salinity stress activated the mitochondrial form within the first 6 h and inhibited the cytosolic form [26]. Previous studies have demonstrated the transcriptional mechanism regulating genes encoding fumarate hydratase isoenzymes in plants exposed to abiotic factors, providing a better understanding of their role in the cellular adaptive response. However, a clear understanding of the mechanism underlying changes in the expression of the studied fumarase genes, including at the level of gene promoter methylation, remains elusive. Studying the epigenetic mechanism regulating the function of fumarate hydratase isoenzymes, particularly changes in the methylation status of individual CpGs in promoters, will allow for a more comprehensive assessment of the role of this enzymatic system in the adaptation of cellular metabolism to stress.

We have demonstrated that light regime and salinity influence the expression of the *Fum1* and *Fum2* genes in sunflower leaves, and hypothesized that these responses may be associated with changes in the methylation status of their promoters. Changes in the methylation status of individual CpGs in the fumarase gene promoters, identified via methylation-specific PCR, may influence the binding of transcription factors to specific *cis*-elements, which are candidate regulatory motifs. NAC transcription factors and light-dependent transcription factors may act as regulators of mitochondrial and cytosolic fumarase gene expression through interactions with putative *cis*-elements, playing an important role in the adaptive response.

## 2. Materials and Methods

### 2.1. Plant Material and Growth Conditions

Leaves of 14-day-old seedlings of sunflower (*Helianthus annuus* L., cv. Flagman) were used in the study. Seeds were germinated on moist filter paper and transferred for hydroponic growth on the second day at 12 h daylight of 90 μmol quanta m^−2^ s^−1^ and a temperature of 25 °C.

### 2.2. Irradiation Experiments

White light was emitted by fluorescent lamps (growth setup Flora-1, PhytoSun, Moscow, Russia). Irradiation by red and far-red light was performed using LEDs with emission regions of 640–680 nm (KIPD40M40-K-P6, Kaskad-Elektro, Moscow, Russia) and 710–750 nm (ZL127A-5, Kaskad-Elektro, Moscow, Russia), respectively. The intensity of red or far-red light during irradiation was 4 μmol quanta m^−2^ s^−1^, and the irradiation lasted 15 min, which was sufficient for the initiation of signal reactions by the phytochrome system but did not lead to the intensification of photosynthesis [27].

### 2.3. Subcellular Localization Studies

The subcellular localization of fumarase was determined using differential centrifugation. The material (5 g) was homogenized with 50 mM Tris–HCl buffer (pH 7.5), 0.4 M sucrose, 5 mM MgCl_2_, 1 mM EDTA, and 5 mM dithiothreitol. The homogenate was centrifuged for 5 min at 1300× *g* and, after discarding the debris pellet, centrifuged again for 20 min at 12,000× *g*. The supernatant was the soluble cytoplasmic fraction. The pellet, containing the fraction of mitochondria and peroxisomes, was disrupted for 15 min by osmotic shock in 1 mL of 50 mM Tris–HCl buffer (pH 7.5) containing 5 mM MgCl_2_, 1 mM EDTA and 5 mM dithiothreitol, and centrifuged for 15 min at 12,000× *g*. Lactate dehydrogenase was used as a cytoplasmic marker and succinate dehydrogenase as a mitochondrial marker [2]. All reagents were purchased from Sigma-Aldrich (St. Louis, MO, USA).

### 2.4. Determination of Fumarase Activity

Plant material (1 g) was homogenized in 5 mL of 100 mM potassium phosphate buffer, pH 7.6, containing 1 mM EDTA, 2 mM dithiothreitol, and 0.01% Tween 80, centrifuged at 12,000× *g* for 10 min, and the supernatant was used for the fumarase assay. Fumarase activity was monitored spectrophotometrically at 240 nm. The unit of activity corresponded to the amount of the enzyme forming 1 μmol fumarate per min. The spectrophotometric medium contained 50 mM Tris–HCl buffer, pH 7.6, 20 mM D,L-malate (sodium salt), 2 mM EDTA, and 5 mM MgCl_2_. The extinction coefficient of fumarate was taken as 2.44 mM^−1^ cm^−1^ [28].

### 2.5. Analysis of Promoter Regions for Salt-Dependent Transcription Factor Binding Sites and Calmodulin Binding Sites

A bioinformatic analysis of the regulatory regions of fumarase genes for transcription factor binding sites was performed using the PlantRegMap/PlantTFDB v5.0 plant transcription factor database (https://planttfdb.gao-lab.org/, accessed on 27 April 2026). Calmodulin (CaM) binding sites within the analyzed sequences were identified using prediction algorithms that use sequence and structural data to predict regions of peptides and proteins that can interact with CaM [29].

### 2.6. RNA Isolation and PCR Analysis

The total cellular RNA was isolated using guanidine–thiocyanate–chloroform extraction using LiCl for sedimentation. RNA concentration in the sample was determined spectrophotometrically. The reverse transcription of RNA was performed using the reverse transcriptase MMULV (SibEnzyme, Novosibirsk, Russia), according to the manufacturer’s recommendations. Real-time PCR was performed using LightCycler 96 (Roche Applied Science, Indianapolis, IN, USA) using SYBR Green for staining. The primers were designed using the software Primer3 [30] (Table 1). Amplification parameters: preliminary denaturation at 95 °C for 5 min, then the following cycle: 95 °C for 30 s, 58–63 °C for 30 s, and 72 °C for 30 s (detection). The amount of matrix was controlled by the parallel amplification of the elongation factor Ef-1 and 18S with gene-specific primers [31]. The stability of expression of these genes under salinity and other stresses has been confirmed by a number of studies [32,33]. The total RNA without the stage of reverse transcription was used as a negative control. Relative levels of expression of the analyzed genes were determined by the 2^−ΔΔCT^ method [34], using the LightCycler 96 Software Version 1.1 (Roche Applied Science, Indianapolis, IN, USA).

For the analysis of gene promoters for the presence of CpG islands and for the design of methylation-specific primers, the program MethPrimer (LiLab, UCSF, San Francisco, CA, USA, https://methprimer.com/, accessed on 27 April 2026) [35] was used; see Table 2 for the full set of methylation-specific primers. The nucleotide sequence of the promoter region of fumarase was obtained from the NCBI database. The polymerase chain reaction with methylation-specific primers was performed using an AmpliSence kit (Helicon, Moscow, Russia). The PCR reaction was performed on a Tertsik instrument (DNA Technology, Protvino, Russia) using the following amplification parameters: initial denaturation at 95 °C for 5 min, then 35 cycles of 95 °C for 20 s, 55–60 °C for 20 s, and 72 °C for 30 s.

The analytical electrophoresis of PCR products was conducted in 1% agarose gel (Helicon, Russia). The results were recorded at 312 nm using a transilluminator and DNA Analyzer (DNA-Technology, Moscow, Russia) and analyzed using the program Gel Explorer, version 1.0 (DNA-Technology, Moscow, Russia). The calculation of quantitative values of methylation-specific PCR was implemented on the basis of electropherograms of PCR products. The semi-quantitative parameters of the degree of promoter methylation represent the total result of PCR analysis of the studied CpG in the promoter of the investigated gene.

Semi-quantitative methylation-specific PCR was performed on the basis of electrophoretic separation of the PCR products. The extent of promoter methylation is an integral index obtained via the PCR analysis of the tested CpG in the promoter of a particular gene. Three types of results could be expected: absence of methylation, partial methylation, or complete methylation. Therefore, we introduced the following definitions: 0% of methylation—all three tested dinucleotides are not methylated; 25% of methylation—1 or 2 CpG are partially methylated, while the rest are completely unmethylated; 50% of methylation—1 or 2 CpG are completely methylated; 75% of methylation—1 or 2 CpG are partially methylated, while other dinucleotides are completely methylated; 100% of methylation—all three dinucleotides are methylated [23]. This evaluation, on the basis of methylation-specific PCR with a semi-quantitative scoring system (0–100%), although limited in resolution, allows for estimation to be made of the degree of methylation of promoters of the corresponding genes [23].

### 2.7. Statistical Analysis

The results are presented as mean values and standard deviations (SD). Statistically significant differences are reported at *p* < 0.05 [36]. The normality of distribution was assessed using the Shapiro–Wilk test. The obtained values allowed us to assess the distribution as normal. A one-way analysis of variance (ANOVA) using Levene’s test showed the homogeneity of the compared groups, with the factor under study exerting a significant influence. Letters represent significant differences according to one-way ANOVA analysis at *p* < 0.05 (Tukey’s multi-comparison).

## 3. Results

### 3.1. Subcellular Localization of Fumarase in Sunflower Leaves

In experiments involving the differential centrifugation of homogenate obtained from 14-day-old sunflower leaves, we separated the cytosolic fraction and the particle fraction enriched by mitochondria. The highest activity of the studied enzyme was observed primarily in the sediment containing mitochondria (75%). The cytosolic fraction contained 25% of fumarase activity (Table 3). Cross-contamination, determined by the activities of succinate dehydrogenase and lactate dehydrogenase, was less than 8% (*n* = 3).

### 3.2. Analysis of CpG Dinucleotides

**Figure 1:**
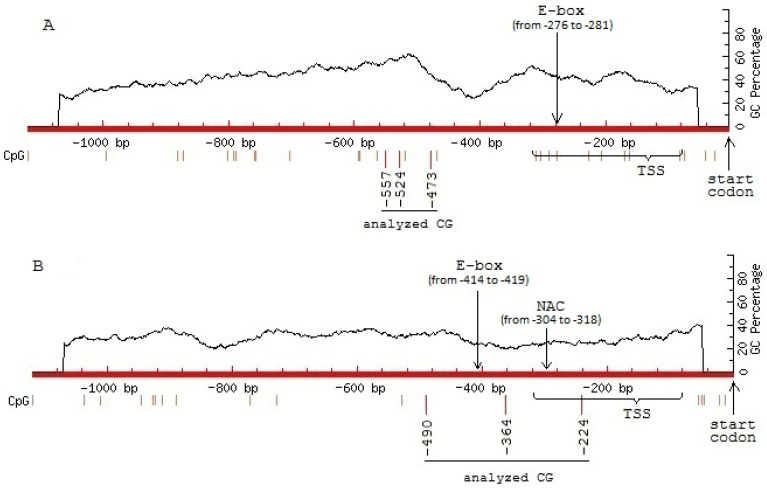
Analysis of CpG in the promoters of the genes *Fum1* (**A**) and *Fum2* (**B**) of *H. annuus* L. Vertical lines show the positions of CpG. TSS—transcription start site. E-box—enhancer box. NAC—transcription factor. The positions of the analyzed *cis*-elements in gene promoters are given in brackets.

The analysis of CpG in the promoters of the genes encoding fumarase was performed using the software MethPrimer (LiLab, UCSF, San Francisco, CA, USA, https://methprimer.com/, accessed on 27 April 2026) (Figure 1). The genes *Fum1* and *Fum2*, encoding, correspondingly, the mitochondrial and the cytosolic fumarase, show a different pattern of CpG, both with the absence of CpG islands. This indicates the tissue-specific standard mechanism of the methylation of CpG, which is not related to the CpG island pattern. The GenBank accession numbers for these genes are XM_022113675.1 (LOC110864574) and XM_022186349.1 (LOC110944702).

An analysis of the nucleotide sequences of the *Fum1* and *Fum2* gene promoters revealed a predicted site of interaction with transcription factors. In the *Fum1* gene promoter, the putative *cis*-element of the phytochrome-dependent factors PIF (E-box) is predicted to be located within the transcription start site (TSS). The *Fum2* gene promoter is predicted to contain two candidate regulatory motifs capable of mediating interaction with transcription factors. The putative *cis*-element of the E-box is located outside the TSS, and the putative binding site for salt-dependent transcription factors NAC is located within the TSS.

### 3.3. Effect of Light on Fumarase Activity and Expression

**Figure 2:**
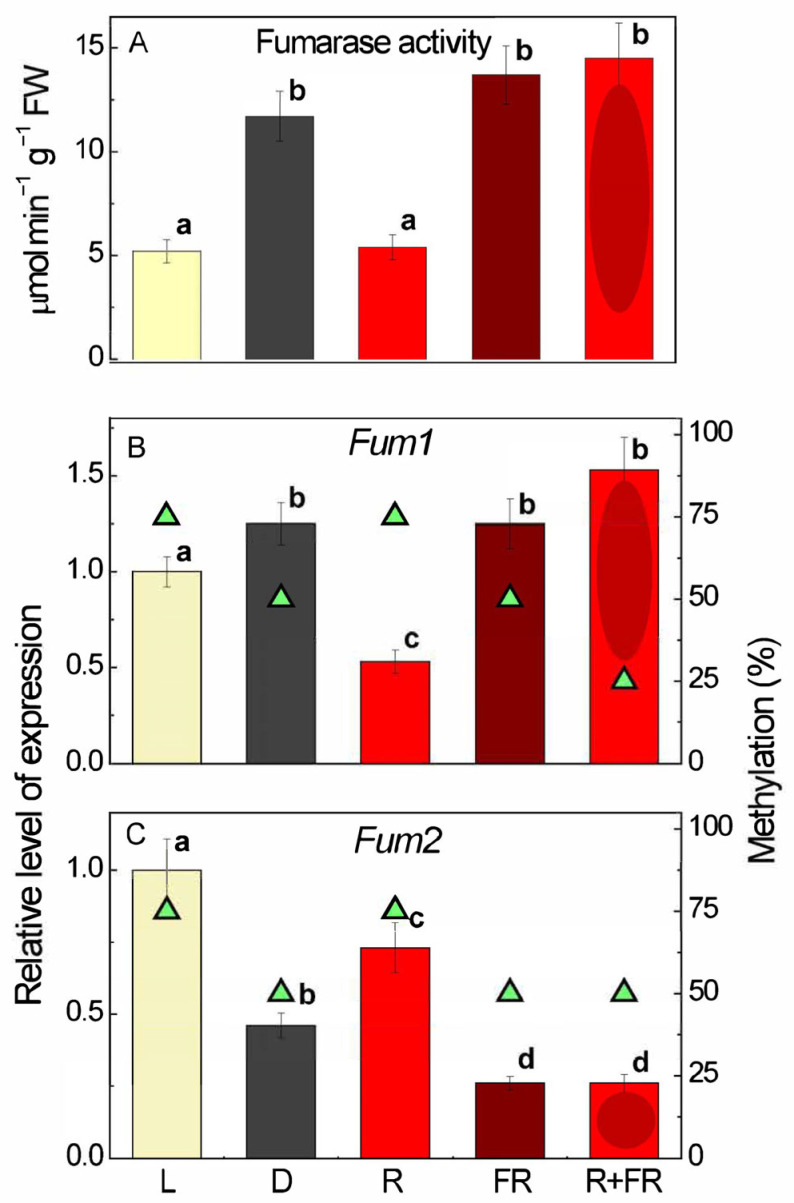
Effect of continuous white light (L), continuous darkness (D), and irradiation by red light (R), far-red light (FR), and far-red light after red light (R + FR) on fumarase activity (**A**), expression of *Fum1* (**B**) and *Fum2* (**C**) genes, and methylation of their promoters (green triangles). The averages (three biological replicates, three technical replicates) ± SD are presented. Letters indicate significant differences compared to control values: *p* < 0.048 for the dark, far-red light, and red plus far-red light, and *p* < 0.046 for red light compared to white light (control). Analysis of variance (ANOVA) was used to process the results.

Fumarase activity was twice as high in darkness than under short-term irradiation by white light and red light, while far-red light applied after darkness or after red light resulted in the return to the activity corresponding to the values in darkness (Figure 2A). The pattern of fumarase activity generally corresponded to the pattern of expression of the *Fum1* gene, which showed smaller differences between the values in darkness and white light, while the inhibition by red light was more pronounced (Figure 2B). Lower Fum1 expression under white and red light corresponded to a higher degree of methylation of the analyzed CpGs in its promoter, as determined by semi-quantitative methylation-specific PCR, suggesting a possible epigenetic effect. Conversely, *Fum2* gene expression was higher under white and red light and lower in the dark and under far-red light (Figure 2C). Higher methylation of the analyzed CpG in the *Fum2* gene promoter corresponded to its higher expression, which is the opposite of the pattern observed for *Fum1*.

### 3.4. Effect of Salt Stress on Fumarase Activity and Expression

**Figure 3:**
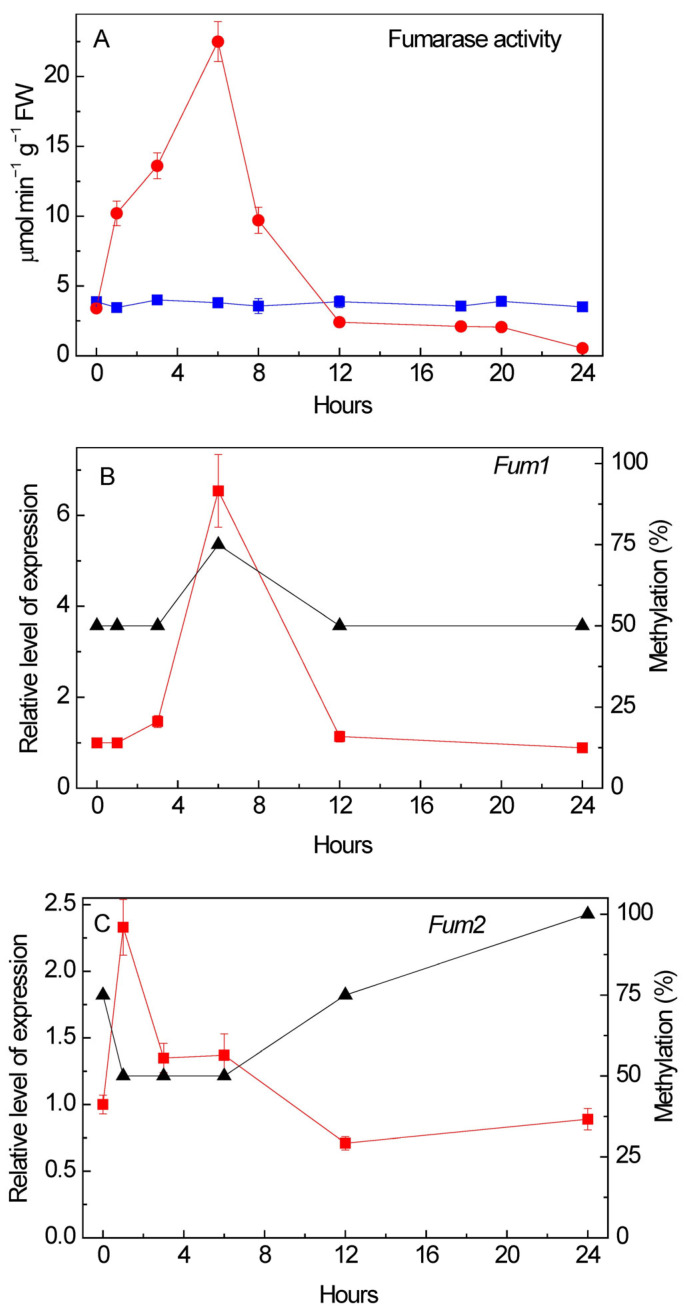
Effect of salt stress on fumarase activity (**A**) (red circles—150 mM NaCl, blue squares—water), expression of *Fum1* (**B**) and *Fum2* (**C**) genes (red squares), and methylation of their promoters (black triangles). The averages (three biological replicates, three technical replicates) ±SD are presented; *p* < 0.040 for fumarase activity, 0.042 for *Fum1* expression and 0.038 for *Fum2* expression compared to control (0 h). Analysis of variance (ANOVA) was used to process the results.

Salt stress (150 mM NaCl) resulted in a fivefold increase in fumarase activity after 6 h incubation, followed by a decline at 12 h below the control level (Figure 3A). The observed increase in activity after 6 h of NaCl exposure corresponded to a sixfold increase in *Fum1* expression and a higher degree of methylation of the analyzed CpGs of its promoter, as determined by methylation-specific PCR (Figure 3B). In contrast, at the early stage of salt stress (1 h), only a twofold increase in *Fum2* expression was observed, followed by a decrease, which corresponded to a higher degree of methylation of the analyzed CpG dinucleotides of the *Fum2* promoter (Figure 3C). For the *Fum2* gene, in contrast to *Fum1*, lower expression may be associated with a higher degree of methylation of the analyzed CpGs, suggesting possible epigenetic regulation.

### 3.5. Bioinformatic Studies of Promoters

**Figure 4:**
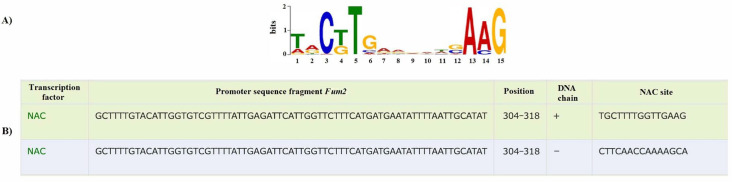
The structure of the binding site of the salt-dependent transcription factor NAC (**A**) and its position in the promoter sequence of the *Fum2* gene of *H. annuus* (**B**).

A bioinformatics study of the nucleotide sequences of the *H. annuus* fumarase isoenzyme gene promoters identified putative *cis*-interaction elements for stress-dependent transcription factors of the NAC family. The predicted NAC binding site was found in the *Fum2* promoter, indicating the possibility of interaction and control of the transcriptional activity of this gene. The predicted NAC binding site is located at positions 304–318 in the “+” direction (sequence TGCTTTTGGTTGAAG) and 304–318 in the “−” direction (sequence CTTCAACCAAAAGCA) (Figure 4). A putative NAC *cis*-element in the *Fum2* gene promoter suggests its role as a regulatory element, which may depend on both the presence of the factor itself and the level of DNA compaction due to its methylation status. Predicted binding sites for other salt-dependent transcription factors of the WRKY family were not detected. No putative NAC or WRKY *cis*-binding elements were detected in the *Fum1* gene promoter.

## 4. Discussion

The existence of two forms of fumarase, one located in the mitochondria and the other in the cytosol, suggests the possibility of their flexible involvement in different metabolic processes. This can be observed in the level of expression of their genes. Even in plants that have one fumarase gene [3], like in yeast [37] and in humans [38], two fumarase forms with different localizations can appear via alternative transcription (alternative splicing) or alternative translation mechanisms.

Species with two fumarase genes encoding the mitochondrial and cytosolic forms should have a mechanism that provides good regulation of expression depending on the metabolic demand of the flux via the TCA cycle in mitochondria, anaplerotic reactions in the cytosol, or divergence to other metabolic pathways. Previously, it was shown that epigenetic modifications in the promoters of fumarase genes could be involved in such regulation [26,39,40]. In the current study, we performed an analysis of fumarase activity, the expression of its two genes, and the methylation of their promoters in response to the conditions of different irradiation parameters and salt stress. The evaluation of the degree of methylation based on methylation-specific PCR with a semi-quantitative scoring system (0–100%), despite its limitations in resolution, allows an estimation to be made of the degree of methylation of promoters of the fumarase genes, similarly to the estimation performed in previous studies [23].

According to the NCBI database, the *H. annuus* genome contains a set of genes encoding a family of DNA methyltransferases. Two genes encoding CpG-dependent DNA methyltransferases were identified: DNM1A (LOC110891067) and DNM1B (LOC110937518). The analysis of the amino acid sequences of these DNA methyltransferases revealed that both enzymes have CaM-binding domains at the C- and N-termini:

DNM1A − 1 [mgtas**l**leseegga**v**kpte] … [algrk**l**keaveakhkt**v**e] 1578;

DNM1B − 1 [mgtas**l**leeaqgaga**v**kpaa] … [algrk**l**keaveakqkq**l**gk] 1584.

The presence of CaM-binding domains at the C- and N-termini ensures high affinity to calmodulin, which allows the Ca-dependent protein to participate in the regulation of its activity [29]. Moreover, different types of interaction sites with calmodulins were demonstrated for two *H. annuus* CpG DNA methyltransferases. The identified CaM-binding sites are of the “1–10 − [FILVW] … [FILVW]” type within the amino acid sequence of DNM1B, and the “1–12 − [FILVW] … [FILVW]” type within the amino acid sequence of DNM1A. The identified features of the amino acid sequences of the analyzed DNA methyltransferases suggest a possibility of their interaction and regulation in a CaM-dependent manner.

Changes in the concentration of free Ca^2+^ in plant cells under different light conditions are controlled by the phytochrome system. Red light causes a phytochrome-dependent increase in calcium content in the nuclei of maize [41] and Arabidopsis [22,42] cells. The presence of the active form of phytochrome in the cell leads to an increase in Ca^2+^ content in the nuclei of maize [41] and Arabidopsis [22,42] cells, which promotes the activation of CaM, converting it into an active complex that regulates the functional state of CaM-dependent proteins. Calmodulins participate in the light regulation of cellular metabolism, mediating the photoreceptor signal at the level of di- and tricarboxylic acid enzymes. As calcium-dependent proteins, calmodulins can control the activity of CaM-dependent enzymes, including DNA methyltransferase.

The observed changes in the mitochondrial form of fumarase, whose expression is greater in darkness and upon irradiation by red light (Figure 2B), are in agreement with the inhibition of the TCA cycle in the light and regulation of its enzymes via phytochrome [43]. According to the data obtained in this study, the inhibition of fumarase can take place through the increase in the methylation of *Fum1* promoters in the light and by red light (Figure 2B), which corresponds to the observed changes in fumarase activity (Figure 2A). The active form of phytochrome exhibits an inhibitory effect on this gene, causing a decrease in its transcript concentration in sunflower leaves when irradiated with red light. This effect of red light is consistent with data obtained for the genes of the TCA cycle enzymes in maize when studying their effects on red and far-red light [39]. In the case of *Fum1*, the decrease in the content of the TCA cycle gene transcripts is due to the inhibition of the entire cycle under conditions of actively functioning photosynthesis [43].

The presence of predicted interaction sites for stress-responsive transcription factors of the NAC family (Figure 4) suggests their role as candidates in the regulation of transcription of these genes under salinity conditions. NAC proteins have a conserved N-terminal DNA-binding domain and a variable transcriptional regulatory region at the C-terminus, which plays a role in either the activation or repression of the transcription of stress-induced genes [44]. This family of transcription factors plays an important role in the plant response to salt stress [45,46]. The presence of a predicted NAC binding site in the promoter of the cytosolic fumarase gene probably facilitates its interaction and may contribute to the regulation of transcription of this gene under salt stress. The probable decrease in the intensity of interaction between the NAC factor and the promoter of the *H. annuus Fum2* gene under salinity conditions for 24 h and the inhibition of its transcriptional activity may be associated with an increase in the methylation status of the analyzed CpG, established by semi-quantitative methylation-specific PCR. The studied CpGs are located in the predicted region of the NAC binding site (Figure 3C); an increase in their methylation status may contribute to an increase in DNA compaction, making it less accessible for interaction with protein factors [47,48].

In the conditions of salt stress (Figure 3), the increase in the expression of *Fum1* corresponds to the phenomenon of “salt respiration”, which represents an adaptive function of respiratory metabolism in response to salinity [49]. It is necessary for the detoxification of toxic Na^+^ and Cl^−^ ions, which requires more energy (ATP) to operate membrane pumps and maintain ion homeostasis, often leading to the increased metabolic activity of mitochondrial processes [50,51]. The high rate of the TCA cycle provides the synthesis of a large number of energy equivalents necessary for cellular biosynthetic processes, aimed at reducing the negative impact of sodium chloride on the plant cell. During this period, the rate of synthesis of osmoprotectors and other metabolites of the adaptive response of cellular metabolism increases significantly [52,53]. The activation of fumarase at the onset of salt stress indicates its participation in metabolic reactions that maintain cellular osmotic balance by regulating the metabolism of vacuolar malate. The mitochondrial form likely contributes most to overall fumarase activity during the first hours of salinization, which correlates with the high expression level of the *Fum1* gene. Based on literature data on “salt respiration,” it is likely that the activation of mitochondrial fumarase we identified contributes to an increase in the rate of TCA cycle function and, consequently, to an increase in the synthesis of energy equivalents, which is necessary for the synthetic processes of osmolyte synthesis and the detoxification of Na^+^ and Cl^−^ ions. This increase lasts only a few hours and, as demonstrated by this study, is not associated with a decrease in the methylation level of the analyzed CpG of the *Fum1* promoter, but rather with an increase in it (Figure 3B). Thus, the methylation of the analyzed CpG of the *Fum1* promoter is unlikely to be a mechanism controlling its activity. The predicted absence of the *cis*-element of the salt-dependent factor NAC in the promoter of this gene likely indicates a different mechanism of its regulation, independent of the CpG methylation status.

For the cytosolic fumarase and its gene *Fum2*, we generally observe the opposite pattern of expression to *Fum1*, depending on light conditions and salt stress. Its expression is higher in the light than in darkness, and it is activated by red light, while far-red light inhibits the expression (Figure 2C). This may be related to the involvement of the cytosolic form in the metabolism of citrate supplied by mitochondria in the cytosol in the presence of light, and in amino acid metabolism [1,6]. This mechanism may play a role in plant cells under conditions of active photosynthesis. A higher expression of *Fum2* is observed with a higher methylation level of the analyzed CpGs of its promoter compared to *Fum1*. Thus, the active form of phytochrome, produced in plant cells irradiated with red light, reduces the overall methylation level of the analyzed CpG dinucleotides of the *Fum2* gene promoter in sunflower. This suggests that either methylation is not directly involved in the regulation of *Fum2* expression by light, or there is an inverse correlation between methylation and gene expression. The two genes studied, *Fum1* and *Fum2*, exhibit opposite responses to light conditions, suggesting that promoter CpG methylation does not fully explain the observed regulation. Other mechanisms regulating the expression of the genes studied may be more important, and promoter methylation may be one element of a common regulatory network.

In conditions of salinity, the increase in *Fum2* expression is smaller than for *Fum1*, and it is only observed at the very beginning of the incubation on salt solution, while at 3–6 h, the expression returns to lower values and then drops below the initial value (Figure 3C). The higher methylation level of the three analyzed CpGs of the *Fum2* promoter, located within the predicted NAC binding site (Figure 1), corresponds to lower expression of this gene. The positive correlation between *Fum2* expression and lower methylation levels of the analyzed CpG dinucleotides of its promoter suggests an epigenetic mechanism for *Fum2* transcriptional regulation. The small number of methylation sites in fumarase promoters and the absence of CpG islands in them (Figure 1) can explain the observed limited role of methylation in the regulation of fumarase activity. The absence of CpG islands suggests the possibility of altering the methylation status of DNA through the methylation of individual CGs, CHGs, and CHHs (where H is A, T, or C), often under the influence of the RNA-directed DNA methylation (RdDM) pathway [54]. Promoters containing a small number of CpGs not organized into CpG islands are characteristic of tissue-specific genes, and the methylation of such promoters does not always correlate with transcriptional repression [55]. The putative E-box-dependent *cis*-element in the promoters of both fumarase genes is located in a region with highly dispersed CpGs (Figure 1), and changes in their methyl status do not affect the formation of a more compact DNA structure and nucleosome.

The methylation pattern, detected by methylation-specific PCR, is restricted mainly to the methylation of *Fum1* via phytochrome and the methylation of *Fum2* in the conditions of salinity, resulting in a decrease in the expression of these genes. Thus, we generally observe the opposite patterns of expression of the genes *Fum1* and *Fum2* depending on light and salinity. The predicted accessibility of *cis*-elements of promoters to transcription factors may play a significant role in this regulation. Increasing the methyl status of the analyzed CpGs located near light-dependent and salt-dependent *cis*-elements promotes the formation and may contribute to a more compact DNA structure [47,48], consequently reducing the accessibility of the promoters of the corresponding fumarase genes to NAC and PIF factors. In the *Fum1* gene promoter, the location of the predicted binding site for phytochrome-dependent factor PIF (E-box) occurs in the TSS, which may play an important role in controlling the transcriptional activity of this gene [56]. For the *Fum2* gene promoter, the two putative *cis*-elements are present: the E-box outside the TSS zone, and the site NAC inside the TSS, which may contribute to its stress-dependent regulation.

**Figure 5:**
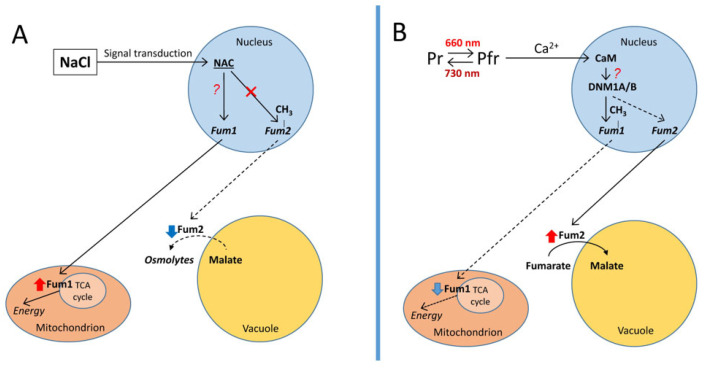
Hypothetical model of possible regulation of the fumarase isoenzyme genes in plant leaves under salt stress (**A**) and light (**B**). Abbreviations: Fum1 and Fum2, fumarase isoenzymes and their genes (italicized); Pr, inactive form of phytochrome; Pfr, active form of phytochrome; TCA, tricarboxylic acid cycle; CaM, calmodulin; DNM1A/B, CpG-DNA methyltransferase; NAC, transcription factor; CH_3_, increase in the methyl status of the promoter. Dashed lines show the decrease in expression of the corresponding genes and the suppression of the associated cellular processes. Red sign × indicates the inhibition of the process. Question marks indicate putative genetic/epigenetic regulatory mechanisms.

Figure 5 presents a summary of the proposed mechanism of regulation of two forms of fumarase by light and salinity, involving the methylation of individual CpG promoters of their genes.

## 5. Conclusions

The obtained results demonstrate that light and salinity are associated with changes in fumarase activity and the differential expression of *Fum1* and *Fum2* in sunflower leaves. These responses coincide with changes in promoter methylation assessed by methylation-specific PCR, suggesting that epigenetic processes may contribute to this regulation. Changes in the methylation status of individual CpGs in the *Fum1* and *Fum2* gene promoters, as determined by semi-quantitative methylation-specific PCR, may suggest the regulation of the interactions of specific transcription factors with predicted *cis*-regulatory elements. Further high-precision methylation and functional studies are needed to establish a causal relationship. Understanding the transcriptional and epigenetic mechanisms regulating metabolic reactions that maintain cellular osmotic balance, using fumarase as an example, will enable the development of a strategy for creating new plant lines that are tolerant to salinity.

## Figures and Tables

**Table 1:** Oligonucleotides for RT-PCR.

Gene	Sequences of Primers
*Fum1*	forward 5′-CCATACCTCTCGCTGAAAGAA-3′
	reverse 5′-ATTGCAGAAGGGTGTGTGTG-3′
*Fum2*	forward 5′-AAATGATCTATTCCCAACCGTGAC-3′
	reverse 5′-TCACAATATTGAAGGGATTAGTAAA-3′

**Table 2:** Oligonucleotides to the promoters of sunflower fumarase genes for methylation-specific PCR.

Gene		Position of the Studied Cytosine	Primer	Sequence
*Fum1*	I	−495 bp	forward M	GGGTGGATTTATATAGAGTGGGTC
			reverse M	ACGAATACCTCATACGAAATAACG
			forward U	GGTGGATTTATATAGAGTGGGTTG
			reverse U	CAAATACCTCATACAAAATAACAAA
	II	−524 bp	forward M	AGGGGTATTACGTGTGGGTTCG
			reverse M	ACGAATACCTCATACGAAATAACG
			forward U	AGGGGTATTACGTGTGGGTTTG
			reverse U	CAAATACCTCATACAAAATAACAAA
	III	−557 bp	forward M	TATTTATGTATAAAATTCG
			reverse M	ACGAATACCTCATACGAAATAACG
			forward U	TATTTATGTATAAAATTTGT
			reverse U	CAAATACCTCATACAAAATAACAAA
*Fum2*	I	−144 bp	forward M	TTTGATTTTTAATTATTGTCG
			reverse M	TGGATTTTTTGTTTTTTGGG
			forward U	TTTGATTTTTAATTATTGTTGA
			reverse U	TGGATTTTTTGTTTTTTGGG
	II	−368 bp	forward M	AATGTATTAAGAAAGTTACG
			reverse M	TGGATTTTTTGTTTTTTGGG
			forward U	AATGTATTAAGAAAGTTATGA
			reverse U	TGGATTTTTTGTTTTTTGGG
	III	−496 bp	forward M	TTATTATATTTTTTGAACG
			reverse M	TGGATTTTTTGTTTTTTGGG
			forward U	TTATTATATTTTTTGAATGG
			reverse U	TGGATTTTTTGTTTTTTGGG

Note: The distance is indicated from the start of the first exon of the gene where the cytosine being studied is located. I, II, and III are different primer groups. Cytosine in each group is either methylated (M) or non-methylated (U).

**Table 3:** Subcellular localization of fumarase in sunflower leaves after separation of cytosol and mitochondria-enriched fraction. Averages from three biological replicates ±SD are presented.

Enzyme	Cytosol Fraction			Mitochondria-Enriched Fraction		
	Total Activity (μmol min^−1^ g^−1^ FW)	Specific Activity (nmol min^−1^ mg^−1^)	%	Total Activity (μmol min^−1^ g^−1^ FW)	Specific Activity (nmol min^−1^ mg^−1^)	%
Fumarase	0.174 ± 0.014	9.10 ± 0.73	25	0.520 ± 0.041	14.2 ± 1.1	75
Lactate dehydrogenase	0.036 ± 0.003	0.20 ± 0.02	92.3	0.0030 ± 0.0002	0.018 ± 0.001	7.7
Succinate dehydrogenase	0.008 ± 0.001	0.115 ± 0.009	7.4	0.10 ± 0.01	0.925 ± 0.074	92.6

## Data Availability

The original contributions presented in this study are included in the article. Further inquiries can be directed to the corresponding author.

## Abbreviations

CaM	calmodulin
PIF	phytochrome interacting factor
TCA cycle	tricarboxylic acid cycle
TSS	transcription start site
